# Effectiveness of a structured teaching program for the improvement of knowledge regarding ethics on health research among members of selected Institutional Review Committees in Nepal

**DOI:** 10.3389/fpubh.2026.1745545

**Published:** 2026-03-16

**Authors:** Namita Ghimire, Richa Acharya, Santoshi Adhikari, Rojina Basnet, Hari Prasad Dhakal, Ramesh Kant Adhikari

**Affiliations:** Nepal Health Research Council, Kathmandu, Nepal

**Keywords:** informed consent, Institutional Review Committee, Nepal, research ethics, training

## Abstract

Members of the Institutional Review Committees (IRCs) were interested in training to effectively carry out their responsibilities to review research proposals for ethical considerations. Thus, an independent educational program was planned to enhance their knowledge of research ethics and familiarize them with ethical research procedures and run the IRC. This study aimed to determine the effectiveness of the educational intervention on knowledge on health research ethics among the members of Institutional Review Committees in Nepal. The study was initiated after obtaining ethical approval. This is a quasi-experimental pretest-posttest study conducted among the members of 11 Institutional Review Committees located in six districts of Nepal from December 2022 to September 2023. The participants went through the structured teaching program consisting of 11 sessions for two days. A validated questionnaire (38 items) was administered before the structured teaching session and post session. The change in the knowledge on research ethics and informed consent process was assessed using McNemar test. Among the 161 participants, nearly a quarter had prior trainings on research ethics including informed consent. The structured teaching program was effective in significantly increasing the knowledge as most of the items had an increase in post-test score compared with the pretest score. The program was successful in increasing in the number of participants who showed improved knowledge regarding ethical aspects of research review process among Institutional Review Committee members (*P* < 0.001). The knowledge on research ethics and informed consent was found to be improved post intervention. The structured teaching program significantly increased knowledge about research ethics and informed consent.

## 1 Introduction

Medical research involving human subjects is on the rise to enhance public health in developing countries (1). The research must adhere to fundamental ethical principles to protect human rights and welfare (2). Numerous research ethics documents, including the Nuremberg code, the Declaration of Helsinki, the Belmont Report and the International Ethical Guidelines for Biomedical Research Involving Human Subjects provide key ethical principles for research involving human subjects (3). These key ethical elements include informed consent, confidentiality, privacy, privileged communication, and respect and responsibility (4). There has been increased awareness of ethical issues due to an increase in number of research (5). The ethics of the research must be clinically justified and scientifically sound (4). International standards mandate that research be reviewed by the research ethics committees (RECs) (6) to ensure adherence to appropriate ethical standards (2). Consequently, all proposals involving human participants require approval from a research ethics committee (7).

The REC, also known as the Institutional Review Board (IRB), plays a crucial role in protecting human research participants through initial and periodic reviews of research proposals. The research ethics committee has an important role to play in ensuring the ethical standards and scientific merit of research involving human subjects (8). Therefore, a thorough knowledge of the ethics of research is mandatory among the research ethics committee members. Proper training is essential, otherwise, the quality of the research reviewed by REC and the RECs' ability to protect the rights and welfare of the research participants may be compromised (9).

The Ethical Review Board (ERB) under Nepal Health Research Council (NHRC) serves as the national REC in Nepal (7). To manage the increasing volume of research (10), the responsibility has been delegated to 60 Institutional Review Committees (IRCs) across the country to provide ethical approval to the growing number of research (11). The members of these IRCs must possess the knowledge and expertise to conduct high-quality review maintaining high ethical standard. The REC members must be competent and trained to evaluate research protocols, ensuring the protection of research participants' safety, rights and welfare (12) while advancing knowledge through rigorous research. Members of the IRCs often interested in training in ethics because of their ethical responsibility for research work. It was therefore felt necessary that there is a need to develop an independent educational program for IRC members to enhance their knowledge and awareness of research ethics and familiarize them with the procedures necessary for ethical conduct in research.

Previous studies show that there is limited evidence on how effective interventions are in improving knowledge of research ethics after they are implemented (13). Therefore, it is crucial to study the effect of the implementation of an educational program on knowledge of health research ethics. In this context, this study aimed to determine the effect of educational intervention on the knowledge of health research ethics among the members of IRCs in Nepal.

## 2 Methodology

### 2.1 Study design and participants

This utilized a quasi-experimental pretest-posttest design in the period between December 2022 and September 2023 to assess changes before and after a structured teaching program among members of 11 IRCs located in Kathmandu, Lalitpur, Kavrepalanchok, Parsa, Dhanusha, and Kaski districts of Nepal. These IRCs were selected based on request letters received for the training. The membership of these IRCs was guided by the National Ethical Guidelines for Health Research in Nepal 2022, with each IRC having 7–15 members. All IRC members were eligible to participate in the study. The members of IRCs included faculty from health sciences disciplines such as Medicine, Pharmacy, Medical Technology, Nursing, and Public Health, as well as laypersons. A self-administered questionnaire was used for data collection.

### 2.2 Study procedures

After obtaining a request letter from the IRCs for the training, a standardized two-day structured teaching workshop was conducted for the respective IRCs. Immediately before participating in the program, a questionnaire titled “Knowledge on ethics in health research” was administered to consented IRC members to assess baseline knowledge.

The training intervention followed a uniform two-day schedule that was applied across all IRCs, with approximately 12 h of contact time. The program consisted of 11 sessions of about 45–60 min each. The same facilitators conducted all workshops using a standardized training package and facilitator guide to ensure consistency of content and delivery. The training was delivered using interactive instructional methods. Each session incorporated facilitated discussion, case-based examples, applied protocol and consent document review, guideline navigation, and participant experience-sharing. Participants worked in small groups during selected sessions to analyze protocol scenarios, identify ethical issues, and discuss appropriate review decisions.

The workshop objectives were to orient IRC members on ethics in health research and the ethical review process, and to strengthen practical skills in reviewing research proposals and consent materials. The lectures followed by discussion was on the following topics:

A. An overview of Nepal Health Research Council and its roles and responsibilities;
B. An overview of Guidelines for Institutional Review Committees (IRCs) for Health Research in Nepal 2016.
C. An Overview of National Ethical Guidelines for Health Research in Nepal 2022.
D. Selection of Appropriate Statistical Methods for Data Analysis.
E. Issue in Protocol Review.
F. Accessing Literature using the Research4life Program.
G. Enhancing Quality of Review.
H. Conduction of REC Meeting, Format of REC Minutes, Format of Standard operating procedures, Documentation and archiving.
Publishing article in a journal.
J. Elements of review for ethical aspects of research.
K. Experience sharing of IRC.

At the end of the second day the “Knowledge on ethics in health research” questionnaire was re-administered immediately to assess the change in the knowledge regarding ethics in health research.

The detailed standardized training schedule are provided in the Supplementary File 1.

The contents of the lectures and the questionnaire for the study were designed after reviewing relevant literatures to better suit a diverse target group, ranging from public health experts to social scientists to laypersons. It was 38-items questions, eight questions related to socio-demographic characteristics of the participants and 30 questions related to knowledge on ethics in health research developed to address the objectives of the study. Content validity of the questionnaire was maintained consulting the literatures and the experts on ethics. The questions ranged from asking about the knowledge on importance of ethics, basic ethical principles, informed consent, amendments approval, good clinical practice (GCP) training, parametric test usage, examples of research misconduct and unethical practices (Supplementary File 2). A multiple-choice format was employed to standardize responses and minimize subjective interpretation, thereby enhancing the reliability of the instrument. The sample size of 161 participants was based on the total number of members across the selected Institutional Review Committees (IRCs), each comprising 7–15 members. All eligible members meeting the inclusion criteria, being an active IRC member at the time of the study and willing to provide informed consent, were invited to participate, ensuring comprehensive representation of IRC members for assessing knowledge of research ethics. Only participants who completed both the pretest and posttest were included in the analyses.

### 2.3 Ethical consideration

This study was conducted in full compliance with the principles outlined in the Declaration of Helsinki. The study was approved by Ethical Review Board of Nepal Health Research Council (Registration number 490/2022). The institutional heads were informed about the study. It was informed that the participation is voluntary, and they can withdraw study at any time without giving reason. Fair explanations were given about the purpose of the study. National Ethical Guidelines for Health Research in Nepal was followed to ensure ethical conduct throughout the research process. Written informed consent was obtained from each participant before administering the questionnaire.

### 2.4 Data analyses

Data were compiled and entered into the Microsoft Excel sheet. The final data sheet was exported to the Statistical Package for Social Sciences (SPSS) version 23 (IBM SPSS Statistics, USA). Participant responses were summarized using frequencies (*n*) and percentages (%). For each questionnaire item, the number of correct responses (*n*) and the corresponding percentage of participants were calculated. The test of normality distribution was done using Kolmogorov-Smirnova-test. McNemar-test was used to compare the individual binary items of knowledge before and after program interventions. *P* value of <0.05 was considered statistically significant. The knowledge scores for ethics and informed consent were combined. Correct answers were coded as 1, while the incorrect answers were coded as 0. The scores were then computed and aggregated. These combined scores were then categorized according to the criteria established in a previous study (13).

## 3 Results

Of the 161 IRC members who participated in the study, nearly one-third of the participants were from Kathmandu district (32.3%), followed by Surkhet (15.5%), Lalitpur (12.4%), Kavrepalanchok (11.8%), Kaski (9.9%), Dhanusha (9.3%), and Parsa (8.7%).

### 3.1 Socio-demographic characteristics of the participants

Table 1 depicts the socio-demographic characteristics of the participants. The mean age of the participants was 39.2 ± 8.9. The majority of the participants were male (55.9%) and most of them were post-graduate qualification (77.6%). Around half of the participants (44.7%) had more than five years of experience in research. Nearly one-third (32.9%) of the participants had more than five publications, while an equal percentage had between one and five publications. Nearly two-third of the participants (64.0%) had received prior training related to research. Among them, 41.6% had received training on research methodology while around one-fourth of the participants (23.6%) had received training on research ethics.

**Table 1:** Socio-demographic characteristics of the participants.

**Variables**	**Frequency (*n*)**	**Percentage (%)**
**Age of the participants**		
Mean ± SD	39.24 ± 8.99	
**Sex**		
Male	90	55.9
Female	71	44.1
**Educational status**		
Bachelor	23	14.3
Postgraduate	125	77.6
PhD and above	13	8.1
**Work experience in research**		
No experience yet	23	14.3
Less than 1 year	20	12.4
1–5 years	46	28.6
More than 5 years	72	44.7
**Publications**		
None	55	34.2
1–5	53	32.9
More than 5	53	32.9
**Prior exposure to trainings**		
Yes	103	64.0
No	58	36.0
**Types of training** ^*^		
Research methodology	67	41.6
Research ethics	38	23.6
Proposal development	34	21.1
Good Clinical Practice	24	14.9
Publication ethics	21	13
Standard operating Procedures	6	3.7
Others	6	3.7

^*^Multiple response.

### 3.2 Changes in knowledge-based responses regarding research ethics

Table 2 shows changes in IRC members' knowledge on research ethics before and after the intervention. Overall, participants' knowledge improved significantly (*P* < 0.05) for most items. Understanding of ethical principles, ERB/IRC approval for amendments, and mandatory GCP training increased markedly. Awareness of procedures such as proposal submission to NHRC and quorum requirements also improved. Some knowledge gaps remained, particularly regarding participant confidentiality (48.4% responded inaccurately) and recognition of research misconduct, including improper use of students' dissertation material (68.3% responded inaccurately). A few areas, such as the need for ethics approval for minimal-risk research, showed slight declines. These results indicate that the intervention effectively enhanced knowledge while highlighting areas requiring further attention.

**Table 2:** Changes in the knowledge-based responses about research ethics before and after program implementation.

**SN**	**Variables**	**Pretest, *n* (%)**	**Posttest, *n* (%)**
1	Importance of research ethics is to protect the right and welfare of the research participants.	130 (80.7%)	133 (82.6%)^***^
2	Autonomy and non-maleficence are basic ethical principles.	44 (27.3%)	83 (51.6%)^***^
3	Investigators, ethics committees and sponsors are responsible for participant protection.	77 (47.8%)	78 (48.4%)^***^
4	There is an ethical guideline for conducting health research in Nepal	130 (80.7%)	140 (87.0%)^***^
5	Number of proposals to be submitted to NHRC before applying for IRC is 10.	97 (60.2%)	133 (82.6%)^***^
6	Quorum fulfillment the means presence of 51 percent of the members in the meeting.	115 (71.4%)	144 (89.4%)^*^
7	Amendment cannot be made without ERB/IRC approval.	44 (27.3%)	131 (81.4%)^***^
8	GCP training is mandatory before conducting clinical research.	13 (8.1%)	145 (90.1%)^**^
9	The principal investigators should have ownership of the data of research.	133 (82.6%)	139 (86.3%)^***^
10	Nominal scale is the weakest level of measurement.	66 (41.0%)	115 (71.4%)^***^
11	Parametric test is used to analyze the data that is normally distributed.	110 (68.3%)	141 (87.6%)^*^
12	Most national and international regulations require ethics committee approval for research studies, even if the research has minimal risks.	100 (62.1%)	89 (55.3%)^***^
13	Some members of the IRC can be unaffiliated with the institution where the IRC operates.	63 (39.1%)	98 (60.9%)^*^
14	All the members of an IRC must have extensive experience in biomedical research and health care.	50 (31.1%)	33 (20.5%)^*^
15	The IRC role includes the consideration of any potential research benefits to the communities where the research will be conducted.	119 (73.9%)	127 (78.9%) NS
16	Protection of participant confidentiality is the responsibility of the researchers and the IRCs.	71 (44.1%)	83 (51.6%)^*^
17	The IRCs must review evidence that the researchers are qualified to conduct the research.	140 (87.0%)	148 (91.9%)^***^
18	The proposals that are less than minimal risk, self-funded, student thesis and single centered can be reviewed by IRCs in Nepal.	61 (37.9%)	123 (76.4%)^*^
19	Faculty taking student's dissertation material for inclusion in own publications without giving due credit is an example of research misconduct.	47 (29.2%)	51 (31.7%)^***^
20	It is unethical if a researcher uses data of an earlier research paper and publishes a new paper.	102 (63.4%)	130 (80.7%)^***^

^*^P < 0.05.

^**^P < 0.001.

^***^P < 0.0001.

NS, non-significant using McNemar test.

### 3.3 Changes in the knowledge-based responses regarding informed consent

Table 3 presents participants' knowledge of informed consent before and after the training program. Overall, there was a statistically significant improvement across most items (*P* < 0.05). Understanding that informed consent must be given by a competent individual after receiving all necessary information increased markedly, as did knowledge of the timing of consent and the review of information sheets by the ethics committee. Awareness of language requirements for consent forms, the legal age for consent, and the need for written assent for children also improved. Some gaps remained, however, particularly regarding retrospective studies, the distinction between the informed consent form and process, and the review of consent documents by a layperson, indicating areas for further reinforcement.

**Table 3:** Changes in the knowledge-based responses about informed consent before and after program implementation.

**SN**	**Variables**	**Pretest, *n* (%)**	**Posttest, *n* (%)**
1	Informed consent is consent given by a competent individual after getting all the necessary information regarding the research.	10 (6.2%)	153 (95.0%)^*^
2	Informed consent is taken before starting the research activity.	138 (85.7%)	146 (90.7%)^*^
3	Consent is not required before conducting the retrospective study.	36 (22.4%)	75 (46.6%)^***^
4	The informed consent form and process are two different processes that complement each other.	140 (87.0%)	148 (91.9%)^*^
5	The information provided in the information sheet should be reviewed and approved by the ethics committee.	130 (80.7%)	143 (88.8%)^***^
6	The legal age for providing consent in Nepal is 18 years and above.	133 (82.6%)	139 (86.3%)^***^
7	The information sheet should be free of scientific and technical terms.	129 (80.1%)	140 (87.0%)^*^
8	In Nepal, the language we should use in the informed consent form is either Nepali or the local language of the participant.	124 (77.0%)	142 (88.2%)^***^
9	In Nepal, children aged 11 to 18 years are required to provide written assent.	73 (45.3%)	87 (54.0%)^***^
10	The informed consent document submitted to ERB/IRC should be reviewed by a lay person.	53 (32.9%)	99 (61.5%)^*^

^*^P < 0.05.

^***^P < 0.0001, using McNemar test.

### 3.4 Effect of educational intervention program on total knowledge scores of the participants

**Figure 1:**
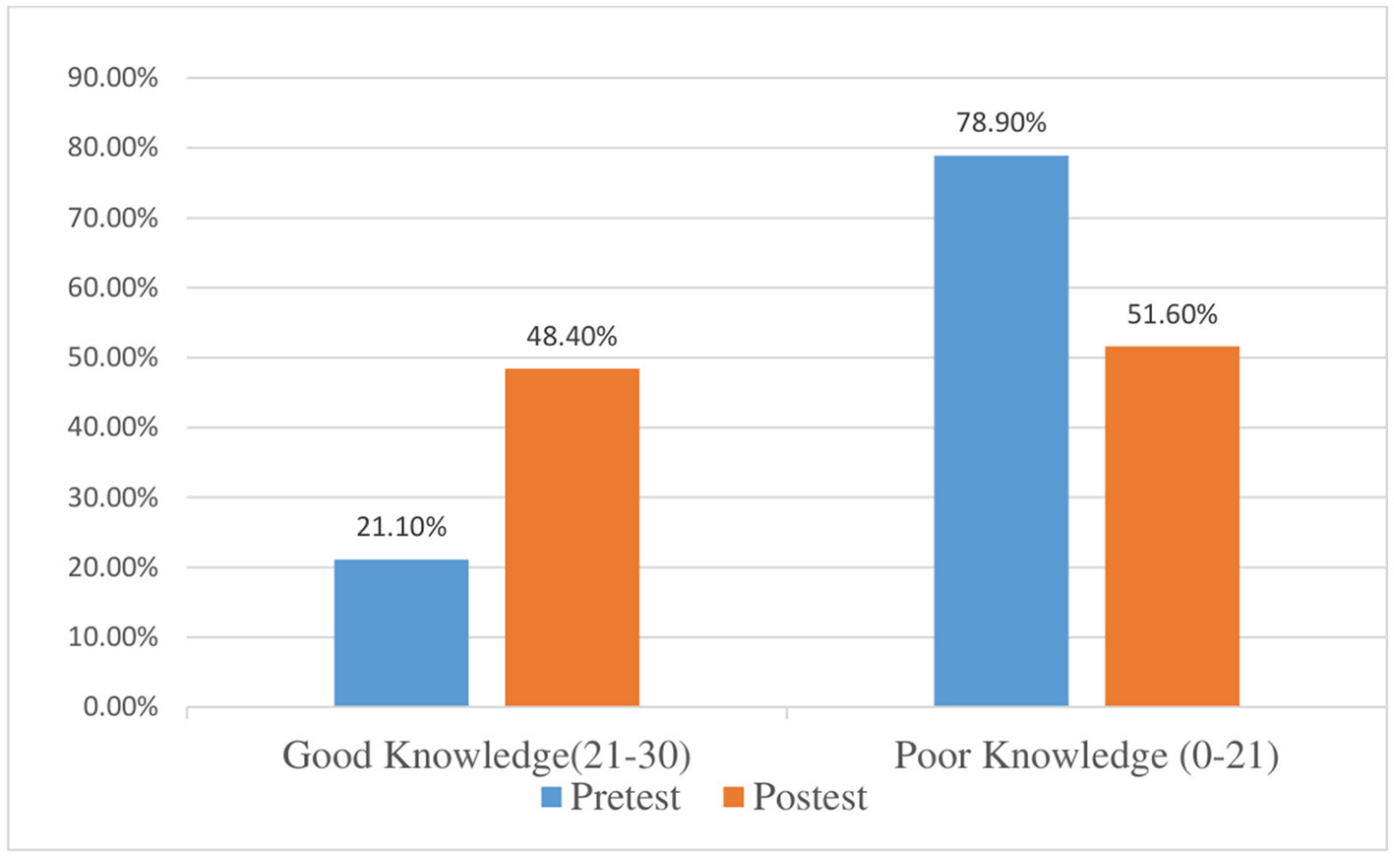
Effect of educational intervention program on total knowledge scores of the participants, pre-and post-intervention.

Participants scoring 70% or less were classified as having poor knowledge, while those scoring above 70% were classified as having good knowledge (Figure 1).

The education intervention program for the participants had a significant increase of mean total knowledge score post-intervention (20.6 ± 3.6) than pre-intervention (18.2 ± 3.7) (*P* < 0.001) as the education intervention was effective in decreasing percentage of poor knowledge from 78.9 to 51.6% post-intervention and increasing percentage of good knowledge from 21.1 pre- intervention to 48.4% post-intervention, and this effect was statistically significant (*P* < 0.001).

### 3.5 Association between participants' sociodemographic characteristics and improvement in knowledge

Table 4 presents the association between participants' sociodemographic characteristics and improvement in knowledge following the research ethics training. Overall, 75.2% of participants demonstrated improvement in knowledge scores after the training. Although a higher proportion of knowledge improvement was observed among participants aged 48–59 years (84.2%) and those holding a PhD or higher degree (84.6%), these associations were not statistically significant. Similarly, no significant differences were observed by sex, educational status, years of experience, number of publications, or prior research-related training (*p* > 0.05). However, a statistically significant association was found between area of expertise and knowledge improvement (*p* = 0.047).

**Table 4:** Association between sociodemographic characteristics and level of knowledge using Chi-square test.

**Characteristics**	Knowledge		***p*-value**
	**Improved (%)**	**Unimproved (%)**	
**Age (in years)**			0.451
24–35	44 (68.8%)	20 (31.25%)	
36–47	57 (78.1%)	16 (21.9%)	
48–59	16 (84.2%)	3 (15.8%)	
60 and above	4 (80.0%)	1 (20.0%)	
**Sex**			0.217
Male	71 (78.9%)	19 (21.1%)	
Female	50 (70.4%)	21 (29.6%)	
**Educational status**			0.604
Bachelor completed	16 (69.6%)	7 (30.4%)	
Post Graduate	94 (75.2%)	31 (24.8%)	
PhD and above	11 (84.6%)	2 (15.4%)	
**Area of expertise**			0.047
Medical doctor	50 (82.0%)	11 (18.0%)	
Public health expert	8 (57.1%)	6 (42.9%)	
Social scientist	4(80.0%)	1(20.0%)	
Biostatistician	3 (75.0%)	1 (25.0%)	
Nurse	15 (55.6%)	12 (44.4%)	
Lawyer	5 (62.5%)	3 (37.5%)	
Others	36 (85.7%)	6 (14.3%)	
**Experience**			0.703
No experience yet	17 (73.9%)	6 (26.1%)	
Less than 1 year	13 (65.0%)	7 (35.0%)	
1–5 years	35 (76.1%)	11 (23.9%)	
More than 5 years	56 (77.8%)	16 (22.2%)	
**Publications**			0.234
1–5	44 (83.0%)	9 (17.0%)	
More than 5	39 (73.6%)	14 (26.4%)	
None	38 (69.1%)	17 (30.9%)	
**Previous research related training**			0.546
Yes	79 (76.7%)	24 (23.3%)	
No	42 (72.4%)	16 (27.6%)	

## 4 Discussion

The study revealed two instances where post-test responses were less accurate than pre-test responses. The belief that most national and international regulations require ethics committee approval decreased from 62.1 to 55.3%, and the belief that all members of an IRC must have experience in biomedical research decreased from 31.1 to 20.5%. This does not mean participants lost knowledge; rather, they revised their earlier understanding after learning new information, which is common in ethics training. Previous evaluations of ethics and integrity training have documented similar phenomena, learners sometimes re-interpret their baseline knowledge after exposure to new concepts, which may temporarily reduce correct responses on straightforward items (14).

The participants' knowledge that GCP training is mandatory before conducting clinical research increased markedly, and understanding that protocol amendments require ERB/IRC approval rose substantially. However, it was seen that for certain critical questions on research ethics, remained incorrectly answered by IRC members. For instance, autonomy and non-maleficence are basic ethical principles, investigators, ethics committee and sponsors are responsible for participant protection, some members of the IRC can be unaffiliated members, the protection of the participant confidentiality is the responsibility of the researchers and the IRCs and the research misconduct. These findings are consistent with prior studies, which report that short-term training often enhances general awareness but may not sufficiently strengthen conceptual or applied understanding of complex ethical principles (15).

Participants' understanding of the concept of informed consent as a process given by a competent individual after receiving complete information showed the largest increases. Similarly, the need for ethics committee review of information sheets and correct use of local language in consent forms also improved significantly. However, IRC members could not answer the key questions on informed consent such that consent is not required before conducting retrospective study and in Nepal, children aged 11 to 18 years are required to provide written assent. These inaccuracies may be due to the delivery method, as information was primarily delivered through lecture sessions. Studies have suggested that adopting small group teaching approaches could potentially enhance learning among IRC members (7, 16).

The study showed that only 23% of the participants had prior research ethics training. This finding contradicts the studies conducted in high-income and middle-income countries (17–19). Since the participants are responsible for regulating the ethics of research projects involving humans, a higher level of research ethics training is essential.

Knowledge regarding the importance of research ethics in protecting participants' rights and welfare increased from 80.7 at pretest to 82.6% at posttest. This finding aligns with the results from a previous cross-sectional study, which also reported a notable level of understanding of ethical principles among participants (20). The increase in knowledge of basic ethical principles in the study is consistent with findings from another study that demonstrated similar improvement (21). More than half of the participants were unaware that the responsibility for participant protection is shared among investigators, ethics committees and sponsors. This contrasts with the review article which emphasizes the importance of investigators REC and sponsors in maintaining ethical standards throughout the research process (22). Despite the ethical guidelines for health research in Nepal, only 87% of respondents answered questions on the presence of these guidelines correctly, similar to the results from a similar cross-sectional study (20).

One-quarter of the participants were unaware that proposals requiring submission to NHRC should number 10, indicating a need for clear communication and guidance to IRC members to ensure they are well-informed about these procedural requirements. Despite the IRC guidelines clearly stating that quorum requires the presence of at least 51% of the members (23), 10% of the participants were still unaware of this requirement. This highlights a significant gap in understanding that could affect the effectiveness of committee operations.

Understanding that protocol amendments require ERB/IRC approval increased markedly after the training, with correct responses rising from 27.3 at pretest to 81.4% at posttest. This increase in understanding underscores the success of the educational interventions carried out during the study. The study findings are consistent with that of another study, which also highlighted the critical requirement for obtaining ERB approval before making amendments (24). Awareness of mandatory GCP training increased, similar to a cross-sectional study where over 80% of participants acknowledged its importance for compliance with ethical and regulatory standards (25).

While most participants identified that principal investigators should own research data, this contrasts with a cross-sectional study where only 37% recognized this role (19). This discrepancy highlights the effectiveness of the educational interventions in raising awareness about the importance of data ownership by principal investigators. The posttest results presented that many participants correctly identified the nominal scale as the weakest level of measurement, consistent with findings from the review article (26). However, when it came to understanding that parametric tests are used to analyze normally distributed data, the posttest results did not show a significant improvement.

Most national and international regulations mandate ethics committee approval for research studies, including minimal risk studies (27), posttest results showed a decline indicating a need for more effective educational strategies to stress the critical importance of obtaining ethics committee approval for all research, regardless of the risk level. More than one- third of the participants were unaware that IRCs include unaffiliated members. This disparity highlights a significant gap in understanding regarding IRC composition among the IRC members. The results revealed a negative outcome regarding the understanding that all members of an IRC must possess extensive experience in biomedical research and health care. This lack of awareness highlights a critical gap in participants' understanding of IRC requirements.

The protection of participant confidentiality is a fundamental responsibility of both researchers and IRCs, as outlined in IRC guidelines (23), was not fully understood by 21.1% of participants even after interventions. The majority of IRC members were aware that they must review evidence of the researchers' qualifications to conduct the research, as stated in the IRC guidelines (23). Despite educational interventions, almost a quarter of the IRC members were still unaware that proposals involving less than minimal risk, self-funded studies, student thesis, and single-centered research can be reviewed by IRCs in Nepal, as stated in the IRC guidelines (27). Additionally, more than two-thirds of the members did not recognize that using a student's dissertation data without giving due credit constitutes research misconduct, which contrasts with the findings of a previous study (24) that showed that 94%−97% of participants were aware of this issue. A quarter of participants were unaware that it is unethical for a researcher to use data from an earlier research paper and publish a new paper, which means crossing into self-plagiarism or salami publication (28).

As stated in the review article (29, 30), informed consent is given by a competent individual after receiving all necessary information about the research. The majority of participants in this study demonstrated an understanding of this concept after interventions. Additionally, understanding that informed consent must be obtained before conducting research activities increased significantly post-interventions. Exempting retrospective studies from informed consent is another item that has been answered correctly by less than half of the participants post- interventions. This is similar to the study (19, 20). Since this study was conducted among IRC members, it is concerning that not all members were aware of this, given their role as the regulatory body. The majority of participants correctly responded that the information provided in the information sheet should be reviewed and approved by the ethics committee. However, more than 10% did not know this, which is concerning given their role as REC members. Despite the practical and everyday exposure of the participants to research ethics as REC members, there was a deficiency in knowledge regarding the age of providing consent for research, even after post-interventions. This is contrary to the cross-sectional study (20), which showed that more than 95% of the participants were aware of this. The discrepancy may be due to the inclusion of a wider range of participants from various backgrounds in the cross-sectional study, who likely had different levels of understanding. More than 10% of the participants had a conflicting understanding regarding the information sheet: it should be free of scientific and technical terms, and the language of informed consent should be in Nepali. In this study, 46% of IRC members were unaware that children aged 11–18 in Nepal must provide written assent, unlike another study (20) where only a quarter knew the correct age. This discrepancy may result from the educational intervention, but it is crucial that all members are aware of this requirement. The national ethical guidelines mandate that informed consent be reviewed by a layperson (27). Despite this, nearly one-third of the participants were unaware of this requirement post-interventions. Further, knowledge can be enhanced through additional REC visits and group discussions.

Regarding the structured teaching program intervention, in the current study, the mean total knowledge score about ethics in research and informed consent pre-intervention was 18.2 ± 3.7. Actually, education intervention study had a significant increase in the mean total scores post-intervention as 20.6 ± 3.6. This effect was highly statistically significant (*P* value <0.001).

The significant association between area of expertise and knowledge improvement suggests that participants' professional background may influence their receptivity to ethics training. Similar findings have been reported in previous studies, where medical and research professionals demonstrated greater gains in ethics knowledge (31). These findings emphasize the need for comparative research to evaluate different educational designs and identify strategies that optimize learning across all professional groups.

Since many participants were busy clinicians, not all were able to attend the pre-survey or post-survey sessions. Consequently, the analyses could not be completed for all training participants, which may have impacted on the results. There are 60 IRCs in Nepal, and this study only includes data from 11 RECs, which may limit the generalizability of the results.

To enhance research ethics and the informed consent process, it is essential to provide additional training workshops and allocate dedicated time for IRCs on research ethics and informed consent. These workshops should include site visits of RECs. Further expanding such types of workshops in other Nepalese IRCs is also necessary. The findings from this study can help shape future workshops, emphasizing skill-building in research ethics and the informed consent process. Additionally, integrating research ethics into medical school curricula could further enrich knowledge in this area.

To our knowledge, this is the first study to assess and enhance the knowledge of research ethics and informed consent among IRC members in Nepal. The study revealed that while there was good baseline knowledge on some aspects of research ethics and informed consent, the structured teaching program significantly improved overall understanding. Nevertheless, some questions were still answered incorrectly after the intervention, indicating that REC members should ideally have demonstrated comprehensive knowledge.

## Data Availability

The original contributions presented in the study are included in the article/Supplementary material, further inquiries can be directed to the corresponding author.
