# Supplementary material for: Effectiveness of a structured teaching program for the improvement of knowledge regarding ethics on health research among members of selected Institutional Review Committees in Nepal
Source: Front Public Health. 2026 Mar 16;14:1745545. doi: 10.3389/fpubh.2026.1745545 (PMC13033679; doi:10.3389/fpubh.2026.1745545)
Supplement: Supplementary file 1 [file Supplementary_file_1.docx]

| **TIDieR Item** | **Description** |
| --- | --- |
| **1. Brief name** | Structured Interactive Training Workshop on Ethics in Health Research for Institutional Review Committee (IRC) Members |
| **2. Why (rationale)** | The intervention was designed to strengthen IRC members’ knowledge and practical skills in research ethics, ethical review processes, informed consent, protocol assessment, and IRC operations, in response to identified training needs and increasing research review responsibilities across Nepal. |
| **3. What (materials)** | Standardized training package including: national ethical guidelines (2022), IRC operational guidelines (2016), SOP and minutes templates, sample research protocols, consent/assent form templates, protocol review checklists, case scenarios, publication ethics examples, and structured facilitator guides. Participants received guideline documents and reference materials. |
| **4. What (procedures)** | A two-day structured workshop with 11 interactive sessions covering: national ethics governance, IRC guidelines, protocol review, informed consent and assent, GCP, statistical considerations in ethical review, research misconduct, publication ethics, REC operations (meetings, SOPs, documentation), literature access tools, and experience-sharing. Activities included case-based discussions, mock protocol review exercises, document critique, small-group problem-solving, and peer experience exchange. |
| **5. Who provided** | Training was delivered by a standardized pool of senior NHRC-affiliated resource persons and ethics experts, including ERB members, IRC accreditation experts, statisticians, and research ethics officers with practical experience in ethical review and IRC operations. |
| **6. How (delivery mode)** | Fully interactive, facilitator-led, delivered through guided group discussion, case-based learning, small-group exercises, applied protocol review tasks, and participant experience-sharing. No lecture-only sessions were used. |
| **7. Where** | Conducted for participating IRCs either in-person at host institutions or via live online workshop format (Zoom), depending on site logistics. Format was consistent in structure and interaction regardless of mode. |
| **8. When and how much** | Delivered over **two consecutive days**, total contact time ≈ 12 hours. Sessions lasted 45–60 minutes each, with scheduled recap, discussion blocks, and feedback sessions. The same schedule was applied across IRC trainings. |
| **9. Tailoring** | Core content and schedule were standardized. Discussion examples and case applications were contextually tailored to participant questions and IRC operational realities, while maintaining fixed learning objectives and topic coverage. |
| **10. Modifications** | No major content modifications were made across sites. Only minor contextual examples varied based on participant background and discussion needs. |
| **11. How well** | A standardized agenda, facilitator guide, and session materials were used across all trainings. The same core facilitator group conducted sessions to maintain consistency. |
| **12. How well (actual fidelity)** | All participating IRC trainings followed the same two-day interactive workshop structure and topic sequence. Formal fidelity scoring was not conducted, but uniform materials, facilitators, and schedules were used. |
